# The importance of leadless pacemaker positioning in relation to subcutaneous implantable cardioverter-defibrillator sensing in completely leadless cardiac resynchronization and defibrillation systems

**DOI:** 10.1016/j.hrcr.2021.06.006

**Published:** 2021-07-02

**Authors:** Mark K. Elliott, Baldeep Singh Sidhu, Vishal S. Mehta, Justin Gould, Dejana Martic, Christopher A. Rinaldi

**Affiliations:** ∗ School of Biomedical Engineering and Imaging Sciences, King’s College London, London, United Kingdom; † Department of Cardiology, Guy’s and St Thomas’ NHS Foundation Trust, London, United Kingdom

**Keywords:** Leadless pacemaker, Subcutaneous ICD, Micra^TM^, Cardiac resynchronization therapy, WiSE-CRT, Endocardial pacing, Heart failure

## Introduction

Leadless pacemakers and subcutaneous implantable cardioverter-defibrillators (S-ICDs) are attractive options to reduce the risks associated with transvenous systems, such as vascular access complications and recurrent lead infections, and their clinical use is increasing.^1^^,^^2^ Leadless cardiac resynchronization therapy (CRT) can be delivered using the WiSE-CRT system (EBR Systems, Sunnyvale, CA), which improves symptoms and left ventricular (LV) remodeling in patients who are untreatable or nonresponders to conventional CRT.^3^^,^^4^ This system uses ultrasound-based wireless communication between a submuscular transmitter and an endocardial electrode to deliver LV pacing, and requires an existing device capable of delivering continuous right ventricular (RV) pacing to achieve CRT. Currently no single-vendor combined leadless system exists to deliver RV pacing, CRT, and ICD therapy. A combination of the Micra leadless pacemaker (Medtronic, Fridely, MN) and the WISE-CRT system has demonstrated the feasibility to deliver leadless CRT^5^ and the addition of an S-ICD (Emblem S-ICD; Boston Scientific, Marlborough, MS) has demonstrated the ability to have a completely leadless CRT defibrillator system.^6^ These devices are not specifically designed to be used together, and co-implantation requires complex programming with the potential for complications related to communication between devices. Here we report the case of a patient with a completely leadless CRT defibrillator system where implant of a new Micra leadless pacemaker resulted in an inappropriate shock from the S-ICD.

## Case report

**Figure 1:**
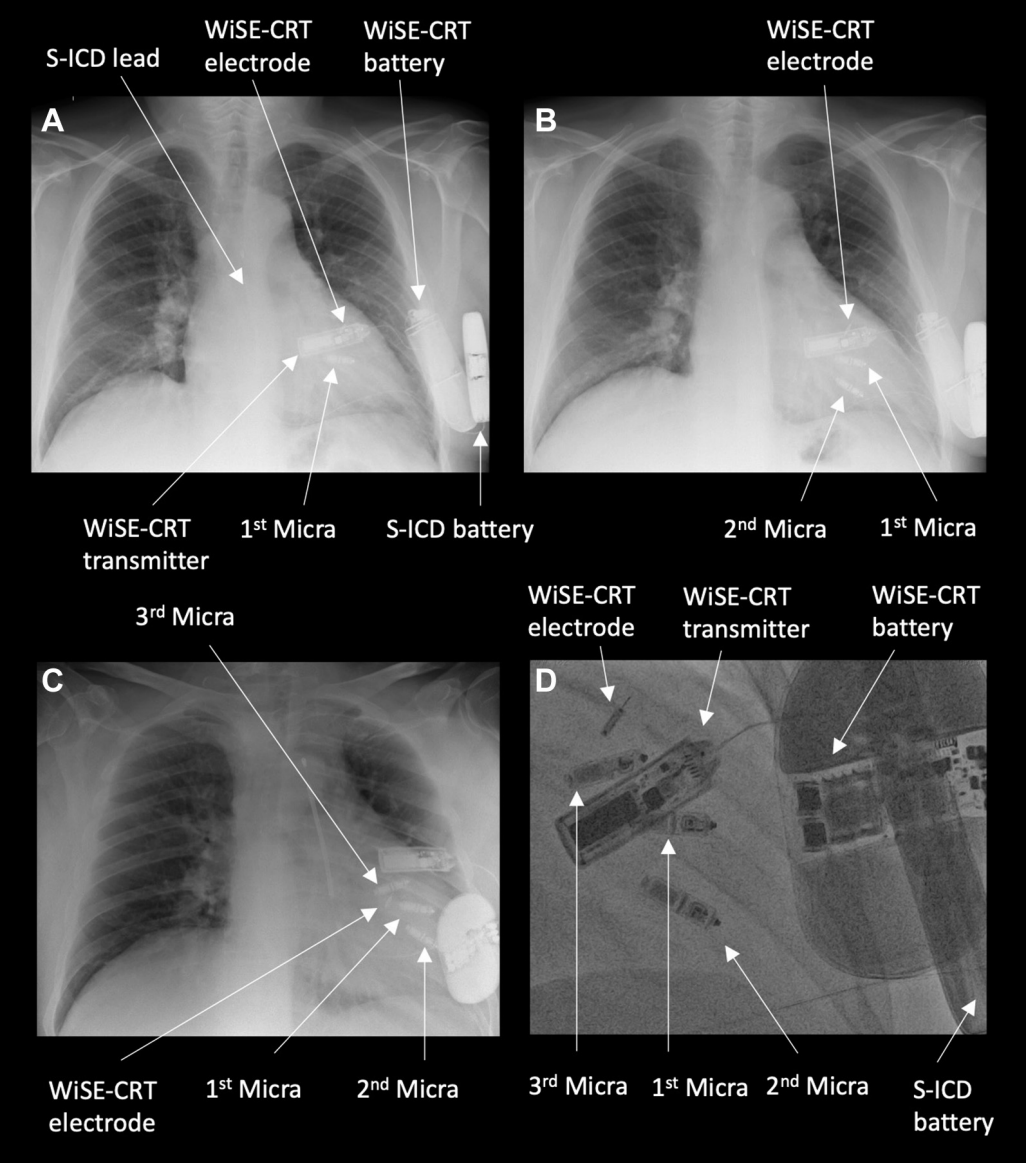
Chest radiographs. **A:** Completely wireless cardiac resynchronization therapy defibrillator (CRT-D) composed of a Micra leadless pacemaker in the right ventricle, a subcutaneous implantable cardioverter-defibrillator (S-ICD), and the WiSE-CRT system. **B:** Addition of a second Micra leadless pacemaker inferior to the original implant. **C:** Addition of a third Micra leadless pacemaker in the right ventricular outflow tract. **D:** Fluoroscopic right anterior oblique view of completely leadless CRT-D system with 3 Micra leadless pacemakers.

**Figure 2:**
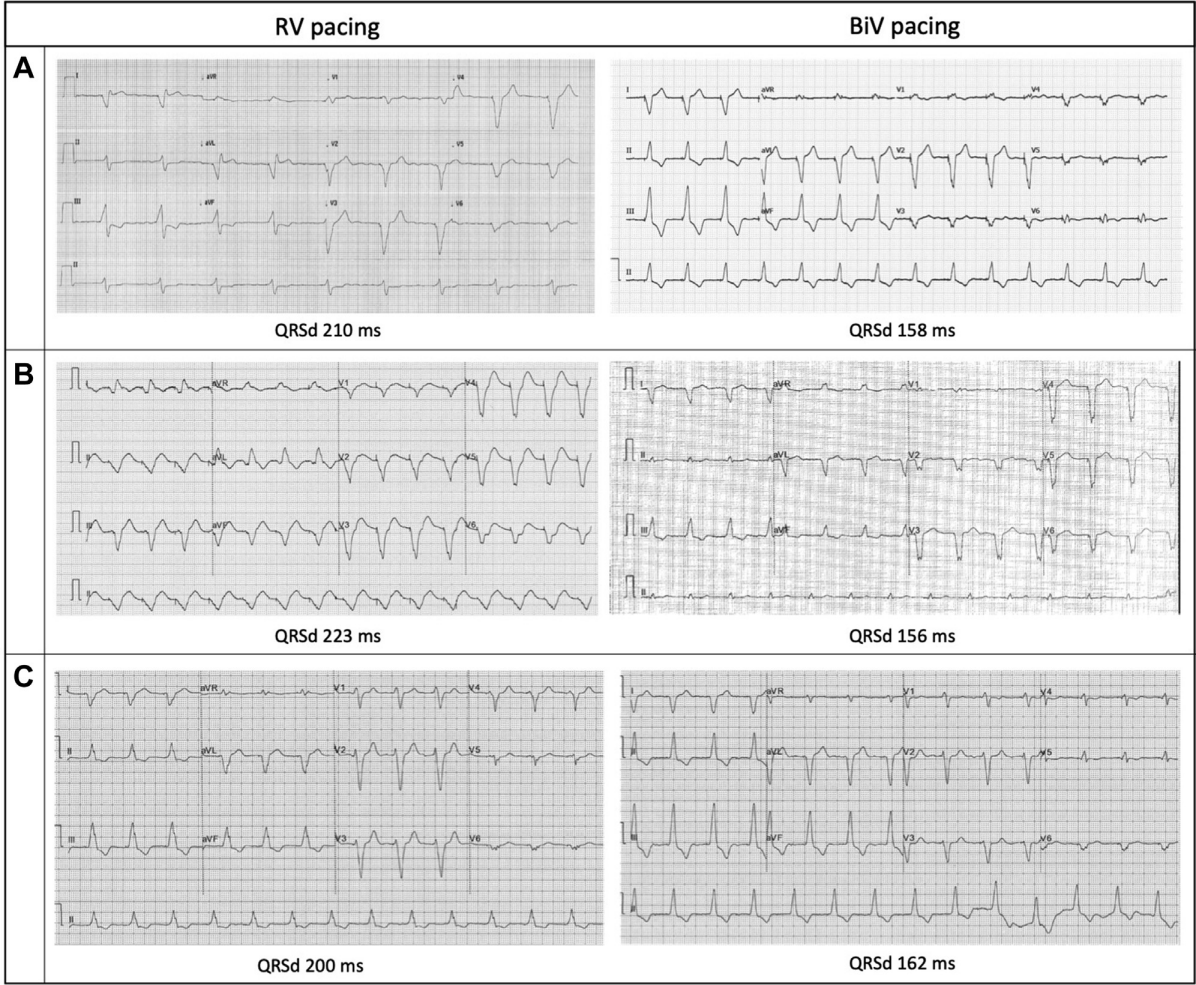
Surface electrocardiograms. **A:** Right ventricular (RV) pacing from initial Micra leadless pacemaker (left) and biventricular (BiV) pacing from the initial Micra and WiSE-CRT system (right). **B:** RV pacing from the second Micra leadless pacemaker (left) and BiV pacing from the second Micra and WiSE-CRT system (right). **C:** RV pacing from the third Micra leadless pacemaker (left) and BiV pacing from the third Micra and WiSE-CRT system (right). QRSd = QRS duration.

**Figure 3:**
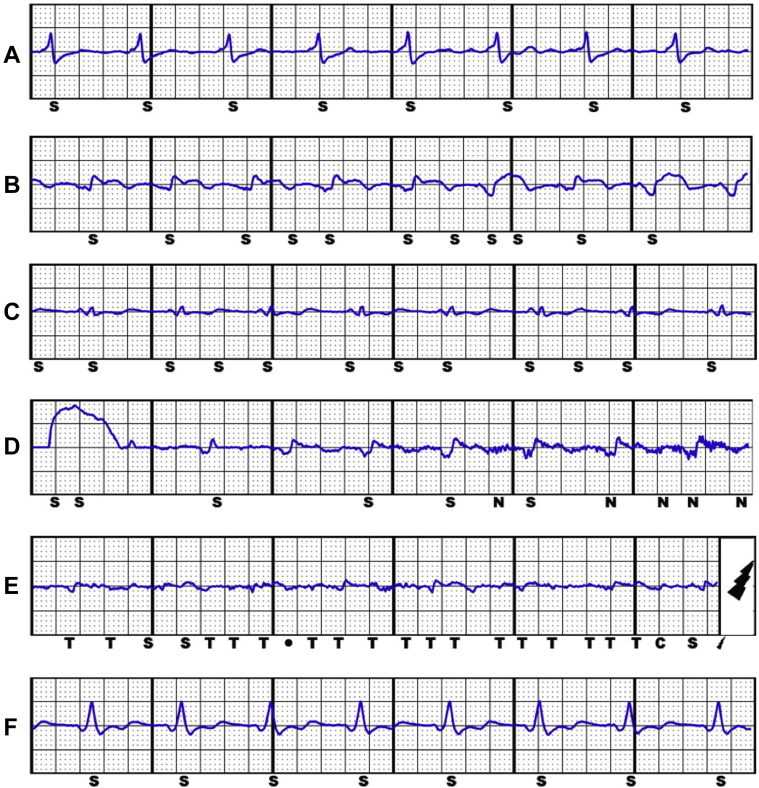
Electrograms from the subcutaneous implantable cardioverter-defibrillator during biventricular pacing from the WiSE-CRT system and different Micra leadless pacemaker positions. **A:** Adequate sensing with initial Micra pacemaker. **B:** Intermittent T-wave oversensing with second Micra pacemaker (alternate vector). **C:** Intermittent T-wave oversensing with second Micra pacemaker (primary vector). **D:** Intermittent R-wave undersensing with second Micra pacemaker (secondary vector) with appropriate sensing of noise. **E:** Small QRS amplitude with second Micra pacemaker (secondary vector) with oversensing of myopotentials and an inappropriate shock. **F:** Adequate sensing with third Micra pacemaker.

A 63-year-old man with a history of ischemic cardiomyopathy, severe LV systolic impairment (ejection fraction 30%), chronic kidney disease, persistent atrial fibrillation, and underlying complete heart block underwent implantation of a completely leadless primary prevention CRT defibrillator system after multiple previous device extractions for recurrent infections, as previously reported.^6^ He was on optimal medical therapy for heart failure including bisoprolol and sacubitril/valsartan, and was anticoagulated with apixaban. The leadless CRT defibrillator system was composed of a Micra transcatheter pacing system in the RV, a WiSE-CRT system, and an Emblem S-ICD (Figure 1A) with effective electrical resynchronization (Figure 2A) and adequate sensing by the S-ICD (Figure 3A). Despite initial excellent pacing parameters, the Micra developed a chronically high pacing threshold (2.75 V at 0.4 ms) and reached elective replacement indicator 17 months after implant. The decision was made to implant a Micra AV device, to allow atrioventricular synchrony during pacing if sinus rhythm was achieved in the future. It was decided not to remove the current Micra, as it had been in situ chronically and the patient had significant LV dysfunction; thus extraction was felt to be high risk. The new Micra AV was implanted inferior to the old Micra device (Figure 1B) with good sensing (R wave 20 mV) and pacing (threshold 0.38 V at 0.24 ms) parameters. During postprocedure programming of the S-ICD, significant T-wave oversensing was noted on the primary and alternate sensing vectors (Figure 3B and 3C), and so the secondary vector was selected. However, it was noted that while there was good sensing of the RV-paced R wave, the biventricular-paced R wave was small in amplitude with intermittent undersensing (Figure 3D). There was reliable tracking by the WiSE-CRT system, with effective electrical resynchronization seen on electrocardiogram (ECG) (Figure 2B). Prior to discharge, the patient experienced an inappropriate shock. Recordings from the S-ICD demonstrated oversensing of myopotentials prior to delivery of the shock (Figure 3E). On further testing, the noise was replicated by movement of the left arm. S-ICD lead position was acceptable on chest radiography (Figure 1B). The S-ICD was reprogrammed to the primary sensing vector, as this minimized the noise; however, this resulted in intermittent T-wave oversensing. During a period of observation there were further episodes of myopotential oversensing detected; however, owing to their intermittent nature, no further shocks were delivered by the device.

In view of the risk of further inappropriate shocks, a decision was made to perform a system revision. Further screening had suggested that repositioning of the S-ICD lead inferiorly would result in acceptable sensing; however, when system revision was planned to be undertaken the patient failed repeat S-ICD screening, with no appropriate vector identified for the current Micra AV / WiSE-CRT combination. It was therefore decided to implant a further Micra AV device in a superior position in the septum / RV outflow tract. Given the patient’s poor LV systolic function and comorbidities, the prior Micra devices were not extracted. In this location there were acceptable sensing (4.3 mV) and pacing (0.5 V at 0.4 ms) parameters and a stable “tug test.” S-ICD sensing with pacing from the new Micra AV location and the WiSE-CRT system was satisfactory (Figure 3F) and the Micra AV was deployed (Figure 1C and 1D). Predischarge checks of the S-ICD and WiSE-CRT system were satisfactory, with reliable biventricular pacing noted, and the patient has had no further myopotential sensing or inappropriate ICD shocks.

## Discussion

This case report demonstrates an important learning point for the management of patients with multiple leadless cardiac devices. While there was appropriate sensing by the S-ICD after initial implant of a Micra leadless pacemaker and WiSE-CRT system, implantation of a new Micra in a different location within the RV changed the biventricular paced QRS morphology, resulting in suboptimal S-ICD sensing. On 2 sensing vectors, T-wave oversensing was noted, which carries a risk of inappropriate shocks. On the remaining sensing vector the biventricular paced QRS complex was low in amplitude, which required an increase in the device sensitivity and resulted in oversensing of myopotentials and an inappropriate shock. Implantation of a third Micra resulted in a significantly different RV and biventricular paced ECG, which resulted in satisfactory S-ICD sensing.

Inappropriate sensing by S-ICD devices can be problematic, with 7%–15% of patients failing initial screening,^7^ and a 7% annual rate of inappropriate shocks reported in an early S-ICD registry.^8^ These were predominantly caused by T-wave oversensing and poor supraventricular tachycardia discrimination, which can be lowered by the addition of the Smart Pass filter^9^ and dual-zone programming,^10^ respectively. The use of the Smart Pass filter in our case was not possible owing to the low amplitude of the paced QRS complex. A recent meta-analysis has demonstrated similar rates of inappropriate shocks between transvenous and subcutaneous ICDs.^11^ Oversensing of extracardiac myopotentials is a less common cause of inappropriate shocks, and is usually inducible during exercise.^12^ Patient education and exercise S-ICD screening may theoretically reduce this risk, though the latter has not been shown to reduce the incidence of inappropriate shocks.^13^ In our case, the myopotential oversensing was due to a fall in paced QRS amplitude, resulting in an automatic increase in the S-ICD sensitivity.

Our case demonstrates that particular care should be taken when a replacement leadless pacemaker is implanted if in combination with an S-ICD, as the resulting change in morphology of the paced QRS complex may cause suboptimal S-ICD sensing. Intraprocedural S-ICD screening is therefore recommended during pacing from the new leadless pacemaker position to ensure adequate sensing prior to deployment. Patients may require the implantation of multiple leadless pacemakers in their lifetime, owing to battery depletion or device failure. The estimated battery longevity of the Micra leadless pacemaker is 12 years^14^; however, this is likely to be significantly shorter for patients who require continuous RV pacing, and for those with high pacing thresholds. Although implantation of multiple Micra devices within the RV is feasible,^15^ our case demonstrates that implanting subsequent devices in a different location may not be optimal in patients with a coexistent S-ICD. Leadless pacemaker implantation sites have previously been chosen on the basis of acceptable pacing and sensing measurements. The requirement to interact with an S-ICD has introduced a new paradigm whereby site selection may also need to take into account the resultant paced ECG morphology to ensure satisfactory S-ICD sensing. Even small changes in device placement may lead to suboptimal ICD sensing, as in this case. Extraction of existing leadless pacemakers is feasible, with a high success rate and low risk of complications,^16^ and may be preferable in such cases, to allow implantation of replacement devices in a similar position to the original. However, myocardial damage at the extraction site may have implications for implantation of a replacement device, including high pacing thresholds or risk of perforation. Further study of extraction and reimplantation of leadless pacemakers is required to assess the safety of this practice, particularly in patients with poor LV systolic function.

## Conclusion

This case highlights the importance of the position of the leadless pacemaker within the RV cavity, as differing paced QRS morphologies may affect S-ICD sensing. This may be an increasingly common and important issue in the future with the increased use of leadless technologies.

**Key Teaching Points**

• A completely leadless cardiac resynchronization and defibrillator system can be achieved using a Micra leadless pacemaker, WiSE-CRT system, and a subcutaneous implantable cardioverter-defibrillator (S-ICD).
• In patients with both a Micra leadless pacemaker and an S-ICD, implantation of a subsequent Micra may change the paced QRS morphology and affect S-ICD sensing, thus highlighting the importance of Micra position within the right ventricular cavity.
• Intraprocedural S-ICD screening to determine adequate sensing from the new Micra position is recommended prior to deployment.
• In such patients, extraction of the existing Micra may be preferable when a replacement device is required, to allow implantation in the same position as the original device, though this may be associated with risks, particularly in patients with left ventricular impairment.
